# The Epidemic of Small Pox in Western New York1Read at the thirty-first annual meeting of the Medical Association of Central New York, at Auburn, October 18, 1898.

**Published:** 1899-01

**Authors:** Edwin R. Bishop

**Affiliations:** Health officer City of Geneva, N. Y.


### THE EPIDEMIC OF SMALL POX IN WESTERN NEW YORK.[1]

BY EDWIN R. BISHOP, M. D., Health officer City of Geneva, N. Y.

THE following facts relating to the epidemic of small pox that visited so many localities in Western New York during the past summer, may be interesting by showing the source of the disease, the difficulty of diagnosis, and the disastrous results from the spread of contagion.

The Joshua Simpkins Opera Company, with twenty-six members, came to Geneva, N. Y., on May 21, 1898, for the purpose of giving

1. Read at the thirty-first annual meeting of the Medical Association of Central New York, at Auburn, October 18, 1898.

an operatic performance. Shortly after their arrival a telephone message was received from the health officer of Ithaca, stating that two days before, while the company was playing in that city, a member of the troupe had been removed from the car, and now showed unmistakable signs of small pox. The troupe was quarantined in their cars, and upon instructions from the State Board of Health the members were vaccinated and the cars disinfected. The disease was evidently contracted in Richmond, Kentucky, early in March.

CASE I.—M. F., never vaccinated, was taken sick in Charleston, W. Va., on March 21st with nausea vomiting and fever, lasting for three days, when a rash appeared on the face, wrists and left foot. The papules became vesicles and then pustules. A physician told him it was chicken pox and he gave up work only during the first stage. His brother, the next case, never vaccinated, became sick fourteen days after contact with the first one, had a similar attack, but with a very profuse rash, and attended to his duties as actor and musician throughout the entire attack. During the pustular stage, while in Fredonia, N. Y., he took a warm bath at the hotel, to allay the pain and itching. While in the water, he pricked all the pustules within reach, allowed the pus to drain out and then wiped himself on the hotel towels, and that evening took the tickets at the theater entrance. This satisfactorily explains the beginning of the very serious epidemic in Fredonia, which resulted in twenty-five cases of the disease, and an inestimable loss of time and money to the inhabitants. The third case was attacked nineteen days after joining the show. His case was also pronounced chicken pox by a physician, and he continued uninterruptedly with his work. The fourth case was much more severe and evidently was true variola. His face was covered with pustules and his body almost as bad, but it was mistaken for chicken pox aggravated by secondary syphilis. He was treated at the hospital for contagious diseases in Ithaca and recovered. The fifth case had been successfully vaccinated some years before. The disease ran a mild course, compelling him to remain in bed during the day for three days, but he took his part as leading man in the play each evening.

When these cases came under our observation, they bore evidences of the rash in small irregular-shaped spots, mostly about the size of a small split pea, with reddened depressed centers, cicatricial in appearance and shedding epithelial scales, the borders brownish, the discoloration fading gradually into the healthy skin. They were all diagnosticated by Dr. Curtis, of the State Board of Health, as small pox. To efficiently quarantine twenty-six persons, many of whom resented their retention and were ready to escape if an opportunity occurred, and in the absence of a hospital for contagious diseases, the local board of health was put to extremities. At first the troupe was quarantined in its traveling cars, but the difficulty of preventing the escape of the men, and the lack of facilities for separating the sick from the well, with the probability that other cases would develop, required more ample quarters, and it was decided to charter the steamer "Onondaga," then lying unused at the dock. Transferring the entire troupe with their effects to her, she was moored at a breakwater in the lake, a sufficient distance from the shore to prevent danger to the townspeople, a corps of attendants and nurses put on board, and communication maintained with the shore by a messenger in a small boat. Provisions were supplied, and I paid one or two visits to the troupe daily as occasion required. The steamer being nearly 200 feet long gave ample quarters for the well persons, a large airy cabin and promenade deck for the sick and nurses, allowing complete isolation, another cabin for suspected cases, besides galleys, dining room, and a furnace in which all the refuse and body discharges were burned. In twenty-four hours a first class quarantine ship was equipped, provisioned and occupied.

All the persons in whom the vaccination was successful were discharged after disinfection in the following manner: Their clothes were thoroughly aired and their bodies bathed for two or three days. On the day of leaving, the clothing and effects were exposed to formaldehyde gas for several hours, their bodies bathed in soap and water and then in bichloride of mercury, 1 to 1,000. They dressed in a clean room and were rowed to shore. Those that were not infected were detained on the boat for two weeks, disinfected in the same way and discharged. The sick ones were detained for some days after the pock marks had ceased to show any scaling, it being considered that all danger was then passed. The steamer was afterward scrubbed, and disinfected with bichloride of mercury, sulphur and formaldehyde gas.

Two cases developed after the quarantine was established:

C. W. C., aged 22, never successfully vaccinated, had fever, nausea, vomiting, malaise and pains in back and limbs. The second day, a papular rash appeared on the forehead and face, extending progressively over his arms, body and legs, involving also the mouth and fauces. On the fourth day, some papules became vesicular with flattened tops and then umbiliated. On the sixth day, many became pustules and by the ninth day the rash was wholly pustular. There was intense tumefaction and congestion of the skin, tissues of the eyes, tongue and throat, causing great pain, closing the eyes and allowing ingestion of liquids only with difficulty. On the tenth day, the pustules began to break, forming confluent crusts that covered the chin and forehead. The primary and secondary fever was marked. Convalescence was very rapid and he was discharged on the thirty-first day quite well, but each pustule left its cicatricial mark, which, however, showed daily improvement. The treatment was symptomatic, and after the first stage, cloths saturated in cold carbolic lotion, 1 in 30, were kept continuously applied to the surface to exclude the light and relieve the pain, and as an antiseptic measure

On the tenth day of the quarantine, E. D., who then had a large umbilicated vesicle on his arm from vaccination, became sick with nausea, malaise fever and pains. In four days he had a well-developed attack of varioloid, with many vesicles, which soon became pustules and then rapidly disappeared, the general symptoms being very mild. He had evidently become infected before being vaccinated, but his attack was anticipated and modified by the vaccination.

The epidemic is interesting and serves to teach us the necessity of great care in the diagnosis of contagious cases. This troupe's course through the state is marked by the trail of disease left behind, in all upwards of 160 cases, and still epidemic in Livingston, Genesee and Tompkins counties. It appears that the disease has existed at McLean in Tompkins county ever since the visit of the company last May. It was thought to be impetigo contagiosum until last week, when its true nature was discovered, but not before twenty-one persons were attacked and hundreds more exposed.

It is, however, a sense of satisfaction to the Board of Health of Geneva to know that the disease was effectually stamped out in the members of the company that came under its care. As far as can be learned, but one case has developed among them, or from them, since their discharge. This man, before reaching Geneva, wrote to his wife in Pennsylvania. She became infected from the letter and had an attack of varioloid. He was successfully vaccinated and discharged from our quarantine, but upon returning to his wife became infected from her, and had varioloid two weeks later himself.

The disease itself seems to have been of a modified form. Most of the cases were mild, even in the unvaccinated, though there were many of true variola. In the troupe of nearly thirty persons living day and night in two cars and exposed to the infection for two months only eleven contracted the disease. One of these carried it to Georgia, where several other persons were infected from him; another was treated in Buffalo, one in Ithaca, one in Pennsylvania, and seven on the quarantine boat in Geneva.

---

MORPHINE DERIVATIVES.—Dresser (*Therapeutische Monatshefte*, No. 9, 1898,) says that heroin produces a stronger sedative action on the breathing than morphine or codeine, over which latter it claims the exceptional advantage that its lethal dose only amounts to one hundred times as much as the ordinary dose. According to Dr. Floret it should be administered with saccharine cough mixture in doses from 0.0005 to 0.01 grammes. Heroin is specially effective in relieving bronchial asthma. The preparation appears to possess no anodyne powers.—*Med. Age.*

